# Neurological consultations via telemedicine for specialized outpatient palliative care (SOPC) at home and in hospice (TANNE project): study protocol for a randomized controlled trial

**DOI:** 10.1186/s12904-022-01088-y

**Published:** 2022-12-06

**Authors:** Shirin Gatter, Kirsten Brukamp, Daniela Adolf, Jürgen Zerth, Stefan Lorenzl, Christiane Weck

**Affiliations:** 1 Research Group Health – Technology – Ethics, Protestant University Ludwigsburg, Paulusweg 6, 71638 Ludwigsburg, Germany; 2 StatConsult Gesellschaft für klinische und Versorgungsforschung mbH, Am Fuchsberg 11, 39112 Magdeburg, Germany; 3 SRH Wilhelm Löhe Hochschule (SRH WLH) University of Applied Sciences, Merkurstraße 19, 90763 Fürth, Germany; 4 Department of Neurology, Hospital Krankenhaus Agatharied, Norbert-Kerkel-Platz, 83734 Hausham, Germany

**Keywords:** Neurology, Palliative care, Telemedicine, Consultations, Study protocol, Partially randomized controlled trial RCT

## Abstract

**Background:**

Neurological diseases cause numerous challenges in palliative care. Telemedicine may improve the access to specialized expertise in neurology for patients, their relatives, and palliative care physicians. The TANNE study offers teleconsultations by a hospital-based neuropalliative center for specialized outpatient palliative care (SOPC) and hospices. A prospective, partially randomized, controlled trial aims at generating evidence for clinical improvements, quality of life, and cost efficiency.

**Methods:**

SOPC and hospice teams in Bavaria, Germany, are partially randomized to one of two study arms, namely a treatment group with teleconsultations by specialists for neurology and palliative medicine or to a control group with interventions after a 12-months delay. Individual and population-based measures are assessed with a mixed-methods design in order to evaluate the medical effects, the potential for implementation in standard care, and health economic aspects. The primary outcome consists of the mean change difference between groups in the Integrated Palliative Care Outcome Scale (IPOS), which physicians assess before and after treatment of a neurological event. Besides, several secondary outcomes are investigated, including quality of life, which is measured with the revised McGill Quality of Life Questionnaire (McGill QOL-R) as well as items regarding general and health-related quality of life. Further secondary outcomes include the concrete progress of the neurological signs and symptoms; the subjective change in well-being since the start of the treatment of the neurological diseases from the perspectives of patients, their relatives, as well as medical and nursing professionals; as well as patient, professional, and caregiver satisfaction with the teleconsultations. Moreover, a health economic evaluation compares group differences regarding hospital visits and emergency calls with utilization measurements.

**Discussion:**

The TANNE trial provides a comprehensive and complex evaluation design for teleconsultations in neuropalliative care. Ethical considerations need to take the patients’ vulnerability into account. The project promises to substantially broaden the width of health care services and to improve the quality of life for deserving patients.

**Trial registration:**

German Clinical Trials Register (www.germanctr.de [July 17, 2022], DRKS ID: DRKS00027436. Registered February 10th, 2022, retrospectively registered. https://www.drks.de/drks_web/navigate.do?navigationId=trial.HTML&TRIAL_ID=DRKS00027436 [July 17, 2022].

## Background

Palliative care aims at improving the quality of life for seriously and terminally ill people. Patients with advanced neurological diseases frequently require palliative care [24, 31], particularly those with neurodegenerative diseases due to a high and complex burden of symptoms [31]. In addition, neurological signs and symptoms provide challenges in oncological palliative states. Oncological patients in palliative care may suffer from confusion and delirium (4 – 88%), depression (20%), anxiety (14%), epileptic seizures (13%), and movement disorders with frequent falls [2, 32, 34]. Consequently, neurological problems and diseases result in increased symptom burden, insecurity [4, 5], and repeated hospital admissions for patients and their relatives, despite appropriate outpatient care services [7].

The need for palliative care due to neurological challenges is expected to rise. Demographic developments predict an increase in the ageing population and thereby in chronic diseases as well as multimorbidity. The historical restriction of palliative care to oncological patients no longer applies (Jess 2019). For many diseases, early access to palliative care is considered meaningful, contributing to the demand for palliative care institutions [27]. Growing social and political pressures on the health care system demand cost-efficient equal access to specialised palliative care for all patients [3]. To ensure this, the use of novel and innovative technologies provides potential solutions. Telemedicine offers the possibility to make expert knowledge accessible to a wide audience. According to a position paper by the Federal Medical Association in Germany [6], telemedicine can supplement conventional patient care, counteract long-term imbalances in health care, and compensate for short-term bottlenecks in services.

In specialized outpatient palliative care (SOPC) teams and hospices, palliative care physicians oftentimes do not possess a continuous access to neurological specialists for instantaneous advice. In order to close this clinical gap, the TANNE project implements a consultation model, which is based on telemedicine, as a new format of care. It investigates and evaluates teleconsultations between palliative care providers and neurological specialists, with participation by patients, relatives as personal caregivers, SOPC and hospice staff. The TANNE project offers access to expertise in neurological and neuropalliative care for patients, their SOPC and hospice teams. An earlier pilot project connected fifteen teams to the telemedical center, demonstrating that meaningful neurological supervision via teleconsultation is feasible. Overall, the care of complex neurological problems in palliative care is supposed to be improved [33].

## Methods/design

### Objectives

The project “Telemedical answers to neuropalliative inquiries in real-time” (i.e. “Telemedizinische Antworten auf Neuropalliative Nachfragen in Echtzeit – TANNE” in German) intends to provide specialist neurological and neuropalliative consultations for patients in palliative care by offering telemedicine and to investigate its benefits scientifically. More specifically, the aims for patients include improvements in symptom control, quality of life, and satisfaction with care (see Table 1). For health care professionals, work satisfaction and confidence in the treatment of complex clinical conditions are supposed to be increased. Furthermore, the utilization of medical resources is compared between treatment and control groups. The TANNE project aims at providing evidence to support an innovative telemedical intervention for a potentially widespread implementation in health care.

**Table 1:** Dimensions and constructs. Three distinct dimensions focus on three bundles of constructs

Dimensions	Focus	Constructs
A	Medical effectiveness	Symptom burden, quality of life, patientsʼ capabilities, change in health state, treatment success, surprise question
B	Potential for implementation in standard care	Confidence with treatment of neurological diseases, satisfaction with treatment overall, satisfaction with care overall, satisfaction with consultation, satisfaction with organization of the consultation, acceptability, caregiver burden, system usability, time requirement
C	Health economic aspects	Internal costs from SOPC and hospice care, external costs like those from hospitalizations and emergency interventions

### Study design overview

Feasibility and effectiveness are evaluated according to three dimensions (see Table 1). Dimension A relates to medical effectiveness. Dimension B examines the potential for implementation in standard care. Dimension C studies the health economic aspects of the intervention.

The study consists of a two-armed, partially cluster-randomized, controlled trial with a delayed start in the control arm (see Table 2). The patients in the treatment arm S1 receive care that includes the innovative intervention. The patients in the control arm S2 get standard care for twelve months and afterwards benefit from the same intervention as the treatment arm S1 for twelve more months. Teams that had already participated in the earlier pilot study [33] were directly assigned to the treatment arm because of their prior experience with the intervention.

**Table 2:** Study arms and phases. Teleconsultation is offered as an intervention in study arm S1 and in phase S2.2 for study arm S2. The study arm S2 in phase S2.1 serves as the control group for the intervention group S1 for the purpose of comparisons between control and intervention groups

Study arms	First year: months 1 to 12	Second year: months 13 to 24
Study arm S1	Phase S1.1: Teleconsultation	Phase S1.2: Teleconsultation
Study arm S2	Phase S2.1: Standard Care	Phase S2.2: Teleconsultation

The study is based on quantitative and qualitative research methods. Questionnaires relying on both validated instruments and newly developed items are provided to all participating teams at different time points to evaluate effects with comparisons between groups and over the course of time (see Tables 3, 4, and 5). Along with this, structured interviews with patients, their relatives, and professionals are conducted, as well as focus group discussions with professionals. Besides, data are included on the patients, their diseases, and their treatments from clinical data management systems, namely Pallidoc (see https://www.pallidoc.de [July 17, 2022]) and the “Information System for Palliative Care” ISPC (i.e. “Informationssystem Palliative Care” in Germany; see https://www.smart-q.de/ed-portfolio/ispc [July 17, 2022]), as examples of documentation systems for SOPC and hospices. A large German statutory health insurance provider (i.e. “Allgemeine Ortskrankenkasse AOK” in Germany) provides financial information on treatment costs.

### Study population, settings, and recruitment

The study is conducted in Bavaria, the second most populated German federal state. The eligible patients to be included for participation either receive SOPC or live in hospices. SOPC is a special form of palliative care tailored to incurable, advanced diseases and a limited life expectancy. Experts from different fields provide care services at home in order to support patients to stay in their familiar environments. Physicians, nurses, and additional health care professionals work together in teams for joint coordination in the patientsʼ familiar surroundings at home and within the family environment.

Patients to be recruited for the study either already suffer from a pre-existing neurological disease, are perceived to likely develop neurological problems, or actually show neurological signs and symptoms. Accordingly, the inclusion criteria are: underlying neurological disease or neurological symptoms, care by an SOPC team or in a hospice, and signed consent form by the patients or their legal representatives. Exclusion criteria are: no neurological symptoms, age below 18 years, pregnancy, residence outside of Bavaria, and no signed consent form. Patients can repeatedly be considered in the study as different cases; new neurological signs and symptoms may be counted as separate events.

The umbrella organizations “State Association SOPC Bavaria” (i.e. “Landesverband SAPV Bayern” in Germany) and “ARGE Hospice – Alliance for outpatient hospice and palliative work in the district of Munich” (i.e. “ARGE Hospiz – Bündnis für ambulante Hospiz- und Palliativarbeit im Landkreis München” in Germany) were contacted and received information on the project. They send out invitations to SOPC teams and hospices. All SOPC teams (i.e. 46 teams) and all hospices (i.e. 17 hospices) in Bavaria are invited to participate in the study.

### Telemedical interventions

Patients in SOPC and hospices receive care by their primary palliative care providers, i.e. physicians and nurses with advanced palliative care knowledge and experience. In addition, as part of the TANNE project, neurologists with a second, additional specialization in neuropalliative care are available as consultants via telemedicine in a regional hospital. Professionals working in the participating SOPC teams and hospices in the treatment arm are invited to use the neuropalliative consultation service for patients who meet the inclusion criteria. A telemedicine kit is located at each participating institution. The kits consist of a tablet computer with a preinstalled app and are equipped with data cards of two mobile network operators. The same intervention implemented in the treatment arm was previously tested and well received in a small, prospective, non-randomized pilot study [33].

### Randomization, study arms and phases

The study is a partially randomized controlled trial (RCT) with two arms, namely a treatment and a control group. The control patients get access to the intervention after half of the study time as well, being part of a delayed start design as an incentive to participate. An allocation ratio of 1:1 is planned for the whole study. Superiority of the treatment versus the control group is supposed to be demonstrated. The study arms and phases are depicted in Table 2.

The participating SOPC teams and hospices are only partially randomized to the arms because some of them already participated in a preliminary proof-of-concept pilot study of the intervention [33]. These teams are all assigned to the treatment arm. Block randomization with random block length is applied for the additional SOPC teams and hospices. Stratification differentiates the type of team, i.e. either SOPC team or hospice. Accordingly, the SOPC teams and hospices are assigned to either the treatment arm S1 or the control arm S2, and all patients in the respective arms are treated equally according to cluster randomization. Randomization is implemented using SAS 9.4 (SAS Institute Inc, Cary, NC, USA) by the StatConsult company. Randomization results are entered into a list of participating teams, after they have been determined and sequentially numbered. The hospital Krankenhaus Agatharied announces allocations of the individual teams only when the sequences of participating teams are fixed. Enrolments of the participating teams are conducted at the hospital Krankenhaus Agatharied, and participating teams recruit patients. All patients of a team are treated according to the team allocation to the treatment or the control arm. As a consequence of the delayed start of the intervention in the control arm and some teams in the preliminary proof-of-concept study, the likelihood to be assigned to the treatment arm is 1 to 3 for the newly participating SOPC teams. For the hospices, the ratio for the assignment is 1 to 2.

### Quantitative study part: primary outcome

The primary endpoint of the study is the difference in symptom burden (see Table 1) before and after treatment of a neurological event, measured by the Integrated Palliative Outcome Scale (IPOS; [22]), assessed by the professionals. The IPOS answers by patients and their relatives are considered by means of a sensitivity analysis. Complementary to the overall IPOS score, intervention effects on individual symptoms are also evaluated in an exploratory fashion. The IPOS consists of nine questions. Two open questions concern the patients’ main problems, which are not covered in the IPOS section with closed questions. The closed questions relate to a list of common signs and symptoms, patient and family distress, patients’ peace of mind, the sharing of feelings with family and friends, information received, and the extent to which practical issues are addressed. The IPOS has been used in various contexts in palliative care, and it has been validated in many languages [22].

### Quantitative study part: secondary outcomes

For dimension A (see Table 1), an important outcome is quality of life assessed by patients. The McGill Quality of Life Questionnaire constitutes a relevant measurement instrument [9], which was specifically developed for palliative care patients. Quality of life is also measured with two single items, namely for general well-being and for health-related quality of life (see Table 4). To assess the treatment quality for neurological symptoms, the subjective change in well-being since treatment start is rated from the perspectives of patients, their relatives as personal caregivers, and the health care professionals. Furthermore, primary palliative care physicians evaluate the concrete development of the neurological signs and symptoms. Besides, the patientsʼ performance status is measured with an integrated scale [30] of the Karnofsky’s index of performance status [15] and the Eastern Cooperative Oncology Group Performance Status Scale (ECOG) [35], rated by professionals. This measure constitutes a standard approach to the assessment of the patient’s health state in palliative care.

With regard to dimension B (see Table 1), a prominent outcome is satisfaction with treatment. The evaluation components tailored towards patients, relatives, and professionals utilize items that relate to the perceived competence and courtesy of the physicians and consultants (see Table 4). The portion on professionalsʼ satisfaction with the consultation contains additional items regarding the consultantsʼ expertise. Furthermore, several items serve to measure the primary physiciansʼ subjective confidence in their treatments and the perceived success concerning neurological signs and symptoms. Other important outcomes are acceptance of the intervention and satisfaction with the organization of the consultation from the perspectives of patients, relatives, and professionals. The estimated durations and the actual time requirements are assessed for the consultation itself as well as for its preparation and wrap-up. Additionally, professionals are asked about satisfaction with palliative care overall. Finally, relatives as caregivers answer questions on their perceived burden due to caregiving.

### Quantitative study part: additional measures

Structural aspects may influence the utilization of the telemedical intervention and thereby the eligibility for standard care. To assess characteristics of the participating teams (see Table 4), professionals answer the Attitudes Toward Health Care Teams Scale [13] and the Internal Participation Scale (TS-6) [17] at the beginning and at the end of the study. Besides, professionals fill in the short scale for technology readiness [23] because they usually initiate the consultations and operate the technological devices. Professionals rate the quality of the technological equipment after the first use with the System Usability Scale (SUS) [12]. Consultants once answer the more comprehensive Computer System Usability Questionnaire (CSUQ) [18].

Furthermore, demographic information is collected from all members of the participating teams (see Table 4). The consultants and the consultants’ office also gather information on the patientsʼ diagnoses, signs, symptoms, treatments, and medication during their consultations with the professionals. This information is analyzed on a qualitative basis due to an expected small number of cases for the individual incidents and diagnoses.

Data from the data management systems Pallidoc and ISPC on the patients, their diseases, signs, symptoms, and treatments are used to control for baseline differences between different populations, namely between the patients in the treatment arm and those in the control arm. Data collected with the telemedicine kit include information on the duration of the consultations. Besides, questions on the connection quality are asked at the end of the telemedical call.

### Qualitative study part

The topics of the qualitative study part cover the effectiveness of the intervention as well as the potential for the implementation of the intervention in standard care (see Table 1). In addition, ethical, legal, and social implications (ELSI) are addressed. Detailed interviews with narrative elements are conducted with select patients, relatives as caregivers, and professionals briefly after the consultation. Focused interviews are planned with patients who are able to verbally communicate shortly after the consultation. Focus group discussions are offered to professionals in the SOPC teams and hospices.

### Health economic measures

To evaluate dimension C (see Table 1), healthcare utilization is compared between the treatment and the control groups. Potential internal (within) costs (i.e. as part of SOPC or hospice care) include coordination of care, consulting services, case management services, care (additive supportive partial care, partial care, or full care), medication, medical aids and appliances. These utilization data are connected with cost weights from AOK billing data and information from the documentation systems PalliDoc and ISPC. To evaluate the internal (within) effects, the incremental changes between costs are calculated with a contingent measurement strategy to compare the distribution of costs between treatment and control groups. Induced or external costs arise outside SOPC or hospices, with a focus on costs due to hospitalizations or emergency interventions, for example treatment in an inpatient palliative care unit, new non-invasive ventilation, or a percutaneous endoscopic gastrostomy (PEG) procedure in the hospital. A major economic effectiveness hypothesis concerns the reduction of hospital admissions due to neurological problems as a consequence of teleconsultations. The relative chance is estimated as 10.6 that a patient in the treatment group is not being hospitalized in comparison to being hospitalized. In addition, dimension C evaluates procedural changes.

### Measurement time points

In the following, the focus is on the quantitative data collection and analysis for dimensions A and B, rather than on qualitative and health economic data. Table 3 illustrates the measurement time points. In particular for dimension A, quantitative data are collected at the start of the study (tS), with the patientsʼ inclusion (tR), between three days and just before the consultation (tB as the baseline measurement time point), directly during the teleconsultation (t0), right after or up to three days after the teleconsultation (t1), three to seven days delayed after the teleconsultation (t2 for the first follow-up), and at the end of the study (tE). If treatment effects are not expected to occur until after t2, another optional measurement time point is specified for 18 to 24 days after the consultation (t21). In the control group, data for t1 are gathered with the occurrence of the neurological issues and close to the contact with the primary palliative care physician without neurological specialization, instead of after the teleconsultation. For t2 and t21, the measurement points are three to seven and 18 to 24 days, respectively, after treatment initiation for the symptoms.

**Table 3:** Study design: time points, participants, measurements, and instruments

**Time point**	**Start**	**Recruitment**	**Baseline**	Teleconsultation*	After teleconsultation*	**Early after event**	Delayed after event**	**End**
**Abbreviation**	**tS**	**tR**	**tB**	**t0**	**t1**	**t2**	t21**	**tE**
**Professionals (in SOPC and hospices)**	• Demographics • Technology readiness • Team collaboration		• IPOS • Karnofsky Index/ECOG • Estimated time requirement*	• Actual time requirement*	• Satisfaction with organization of consultation* • Usability*	• IPOS • Karnofsky Index/ECOG • Change in health state • Confidence with treatment • Satisfaction with consultation* • Treatment success for neurological disease* • Acceptability* • Satisfaction with palliative care	Like t2	Like tS
**Consultants**	• Demographics • Technology readiness • Usability		• Estimated time requirement*	• Actual time requirement*	• Satisfaction with organization of consultation* • Surprise question* • Technical aspects*		Like t1*	Like tS
**Patients**			• Demographics • IPOS • QOL			• IPOS • QOL • Change in health state • Satisfaction with treatment • Satisfaction with consultation* • Acceptability*	• IPOS • QOL	
**Relatives (as caregivers)**			• Demographics • IPOS • Caregiver burden			• IPOS • Change in health state • Satisfaction with treatment • Satisfaction with consultation* • Acceptability* • Caregiver burden	Like t2	

* Teleconsultations, with the consultants’ involvement, only occur for S1.1, not S2.1, during the first year. ** Optional time point in case of expected late treatment effects

### Measurement instruments

Besides standard instruments, self-developed scales are utilized for quantitative aspects in dimensions A and B, construed from own items or items that are, to different degrees, inspired by the literature (see Table 4). Some items are translated and directly adopted from other scales. Items may be taken from existing scales and modified for the study, e.g. regarding expressions. Additional items are loosely inspired by a variety of scales, such that the corresponding items capture the same idea as the original items, but the phrasing is different. Table 4 lists the measurement instruments with their item sources. Table 5 provides a summary of the measurements per participating group, the measurement time points, and the principles of analysis.

**Table 4:** Instruments and item sources. The term “professionals” refers to health care providers in SOPC and hospices, including primary palliative care physicians, but not including consultants for specialized neuropalliative care, who are designated as “consultants”

Constructs	Instruments and item sources	Number of items	Target groups
Sociodemographic data	Own items	2	Patients
	Own items	7	Relatives
	Own items	6	Professionals (physicians)
	Own items	6	Professionals (nurses)
	Own items	7	Consultants
	Own items	5	SOPC and hospice teams
Symptom burden	Integrated Palliative Care Outcome Scale IPOS [22]	17	Professionals, patients, relatives
Quality of life	McGill Quality of Life Questionnaire McGill QOL-R [9]	15	Patients
Quality of life – overall	Item taken from [10]	1	Patients
Quality of life – health-related	Item modified from the EQ5D/EuroQol [29]	1	Patients
Patientsʼ capabilities	Karnofsky Index/ECOG [15, 35]	1	Professionals
Caregiver burden	Items taken and partly modified from the Quality of Life in Life Threatening Illness-Family Carer Version Questionnaire QOLLTI-F [8]	5	Relatives
Change in health state	Own item: change in health state since treatment of neurological signs and symptoms	1	Professionals, consultants, patients, relatives
Change in health state and neurological state	Own items: change in health state as well as neurological signs and symptoms since treatment of neurological signs and symptoms	2	Professionals (via consultantsʼ office)
Confidence with treatment	Own items: confidence with treatment of neurological diseases	5	Professionals
Satisfaction with treatment overall	Items modified from the Telehealth Satisfaction Scale TESS [1, 20, 21, 28] and own items	10	Patients, relatives
Satisfaction with consultation	Items modified from the TESS [11, 21] and own items	7	Professionals
Success of treatment of neurological diseases	Items inspired by [11]	2	Professionals
Satisfaction with organization of the consultation	Items inspired by the Internal Participation Scale IPS [14, 17] and own items	3	Patients
		6	Relatives
		4	Professionals
	Items inspired by the IPS [11, 14, 17] and own items	5	Consultants
Surprise question	Surprise in case of death [26]	1	Consultants
Acceptability	Items inspired by the eHealth literacy questionnaire eHLQ [16] and the Telehealth Usability Questionnaire TUQ [25]	2	Professionals, patients, relatives
Satisfaction with palliative care overall	Items inspired by [14] and own items	3	Professionals
Estimated and actual time requirement	Items inspired by [19]	6	Professionals, consultants
Technical aspects of consultation	Items inspired by the TUQ [25] and the Post-Study System Usability Questionnaire PSSUQ (Lewis 2002)	4	Consultants
Technology readiness	Short Scale for Technology Commitment [23]	12	Professionals, consultants
Team participation	IPS [17]	6	Professionals
Attitude toward teamwork in healthcare	Attitude Toward Teamwork in Healthcare Scale [13]	19	Professionals
System usability	System Usability Scale SUS [12]	10	Professionals, consultants
	Computer System Usability Questionnaire CSUQ [18]	19	Consultants

**Table 5:** Study arms, measurement time points, and principles of analysis. The type of analysis depends on the data available. The term “vs.” (i.e. “versus”) indicates comparisons between groups. * denotes optional time points dependent on the clinical course

Construct	Target groups	Study arms	Measurement time points	Principles of analysis
Sociodemographic data	Patients	S1, S2	tB	Descriptive
	Relatives	S1, S2	tB	Descriptive
	Professionals (physicians)	S1, S2	tS, tE	Descriptive
	Professionals (nurses)	S1, S2	t2	Descriptive
	Consultants	S1, S2.2	tS, tE	Descriptive
	SOPC and hospice teams	S1, S2	tS	Descriptive
Symptom burden	Professionals, patients, relatives	S1, S2	tB, t2, t21*	S1 vs. S2 tB vs. t2
Quality of life	Patients	S1, S2	tB, t2, t21*	S1 vs. S2 tB vs. t2
Quality of life – overall	Patients	S1, S2	tB, t2, t21*	S1 vs. S2 tB vs. t2
Quality of life – health-related	Patients	S1, S2	tB, t2, t21*	S1 vs. S2 tB vs. t2
Patientsʼ capabilities	Professionals	S1, S2	tB, t2, t21*	S1 vs. S2 tB vs. t2
Caregiver burden	Relatives	S1, S2	tB, t2, t21*	S1 vs. S2 tB vs. t2
Change in health state	Professionals, consultants, relatives	S1, S2	t2, t21*	S1 vs. S2
	Patients		t2	S1 vs. S2
Change in health state and neurological state	Professionals (via consultantsʼ office)	S1, S2	t2, t21*	S1 vs. S2
Confidence with treatment	Professionals	S1, S2	t2, t21*	S1 vs. S2
Satisfaction with treatment overall	Patients, relatives	S1, S2	t2	S1 vs. S2
Satisfaction with consultation	Professionals	S1, S2.2	t2, t21*	Descriptive
Success of treatment of neurological diseases	Professionals	S1, S2.2	t2, t21*	Descriptive
Satisfaction with organization of the consultation	Patients	S1, S2.2	t2	Descriptive
	Relatives	S1, S2.2	t2, t21*	Descriptive
	Professionals	S1, S2.2	t1, t21*	Descriptive
	Consultants	S1, S2.2	t2, t21*	Descriptive
Surprise question	Consultants	S1, S2.2	t1	Descriptive
Acceptability	Professionals, patients, relatives	S1, S2.2	t2, t21*	Descriptive
Satisfaction with palliative care overall	Professionals	S1, S2	t2, t21*	S1 vs. S2
Estimated and actual time requirement	Professionals, consultants	S1, S2.2	tB, t0	tB vs. t0
Technical aspects of consultation	Consultants	S1, S2.2	t1	Descriptive
Technology readiness	Professionals, consultants	S1, S2	tS, tE	tS vs. tE Moderation
Team participation	Professionals	S1, S2	tS, tE	tS vs. tE Moderation
Attitude toward teamwork in healthcare	Professionals	S1, S2	tS, tE	tS vs. tE Moderation
System Usability	Professionals	S1, S2.2	t1	Descriptive
	Consultants	S1, S2.2	tS, tE	Descriptive

### Sample size calculation

According to a validation study for the IPOS [22], the minimal relevant difference is 4.3 points, with a standard deviation of 9.3 points, and this difference is here assumed as the one between the intervention arm and the standard treatment arm. Using a significance level of 5 percent and a desirable power of 80 percent (via Proc Power, SAS 9.4, two-sample t-test for mean difference), 75 patients with neurological diseases need to be included in the treatment arm S1 as well as in the control arm S2.1 in order to show the assumed difference.

### Statistical data analysis

Several analysis populations are distinguished for the evaluation. The full-analysis set A (FAS-A) contains all included patients, for whom baseline data are available. The full-analysis set C (FAS-C) contains the same patients, but is potentially extended by a virtual control group for the evaluation of dimension C. Evaluation-set A (ES-A) includes all FAS-A patients who actually experience a neurological problem during the study. Partial-evaluation set A (tES-A) includes all FAS-A patients who do not experience a neurological problem during the study. An investigation of the different analysis sets for dimension A (FAS-A, ES-A, and tES-A) allows a control for a possible bias with regard to the baseline data. No imputation of missing data is planned. Quantitative data are analyzed with the current versions of the statistical software packages SPSS, SAS, and R.

The intervention effectiveness is analyzed by comparisons between groups and within groups. In Table 5, the intended analysis is described for every outcome. Comparisons between the control and the treatment group are conducted when data from both groups are available. When data from measures before treatment start are simultaneously available, they are considered in the same analysis to control for previous differences between the groups.

For the primary outcome according to IPOS, the difference in the mean change from baseline (tB) to the follow-up (t2) is analyzed between groups with tests for homogeneity. The same applies to the quality of life measures, the Karnofsky Index/ECOG, and the caregiver burden. For these measurements, data are available from two points in time.

Tests for homogeneity of differences between groups are conducted for the perceived change in health state, overall satisfaction with the treatment according to patients and their relatives, confidence with the treatment of neurological diseases, and satisfaction with palliative care overall. These data are provided by both the intervention arm and the control arm.

Homogeneity tests for differences are not possible for satisfaction with the consultation, success of neurological treatment, satisfaction with the organization of the consultation, overall satisfaction with the treatment according to professionals, and acceptability of the teleconsultations. Instead, descriptive statistics are reported.

Furthermore, comparisons between early and late study phases in the intervention group (i.e. between S1.1 and S1.2; see Table 2) allow to investigate consequences of repeated use, familiarity, and learning due to previous experiences with telemedicine and the accompanying activities, such as optional educational online seminars and communication between care providers.

Structural information on the teams is collected at the start of the study. For example, technological readiness, team participation, and attitude toward teamwork may influence the utilization of the intervention and its effectiveness. These measures are therefore considered as moderating factors. Besides, they provide data on the specific characteristics of the participating teams, allowing conclusions on the generalizability of the results. In addition, this information is assessed at the end of the study to investigate whether technological readiness or team participation change over the course of the study.

The primary endpoint, i.e. the difference in the mean change of the IPOS from baseline (tB) to the follow-up time point (t2), is analyzed between groups with tests for homogeneity. A mixed linear model is used in order to include potential confounders as well as several potential events per patient and to account for the patientsʼ cluster randomization. Tests for homogeneity of differences between groups will also be conducted for most secondary outcomes, i.e. quality of life measures, Karnofsky Index/ECOG, caregiver burden, perceived change in health state, overall satisfaction with the treatment according to patients and their relatives, confidence with the treatment of neurological diseases, and satisfaction with palliative care overall.

A health economic evaluation according to a cost–benefit approach is conducted to address the differences in induced health care effects, especially hospital admissions that interrupt patients’ outpatient care periods. Differences are assessed regarding the number of hospital admissions and cost-weights of each induced hospitalization, in comparison between groups. Using a sequential approach, hospital admissions are identified that can be connected to neurological problems. For this purpose, matched pair analysis methods are applied, using a virtual control group. In the health economic study part, patients with an event, such as induced hospital admissions, are evaluated as a subgroup within the corresponding treatment arm.

### Project organization and funding

As part of the TANNE project, the clinical trial is financed by the Innovation Funds of the Federal Joint Committee for health care in Germany. The Committee promotes new formats of health services for future development and innovation. The specialist center for neurology and neuropalliative care offering consultations is located at the medium-sized hospital Krankenhaus Agatharied, in Hausham, Bavaria, an academic teaching hospital of the Ludwig-Maximilians-University LMU Munich. The department of neurology organizes and leads the project, the teleconsultations, and the collection of new clinical data. The study center at the hospital Krankenhaus Agatharied continuously monitors the protection of the rights and the safety of the study subjects, the completeness and verifiability of the study data, and the compliance of the study with the protocol, good clinical practice, and applicable regulatory requirements. Members of the research group Health – Technology – Ethics at the Protestant University Ludwigsburg primarily evaluate dimensions A and B. The ISO13485-certified company StatConsult is responsible for the statistical analysis and the data management. Members of the research group International DiaLog College and Research Institute IDC at the SRH Wilhelm Löhe University of Applied Sciences Fürth, together with the department of economics at the University of Bayreuth, especially evaluate dimension C. The company MEYTEC provides the technological equipment and the technical support for teleconsultations. The AOK Bavaria supports the project with special contracts for services outside the scope of standard care, by providing routine insurance data for the evaluation, and by consulting from a financial perspective. The AOK insures 38 to 51% of patients in SOPC. Furthermore, on average, the AOK pays for 50% of the billing cases in SOPC (Bundesministerium für Gesundheit 2019).

## Discussion

### Relevance

Telemedical services promise sustainable improvements in health care, both regarding general outcome measures and individual satisfaction. The TANNE study aims at generating the evidence by a prospective, partially randomized, controlled trial. If the expectations are met, health care will be transformed because of increased insurance support for a novel model of interventions with teleconsultations in palliative care, since a large German statutory health insurance provider is part of the consortium.

The project TANNE addresses the highly specialized field of neuropalliative care, namely by expert neurological advice for palliative care as SOPC at home and in hospice. Thereby, the clinical access to rare expertise is strengthened for patients, relatives, and primary physicians in general palliative care alike. Teleconsultations improve information exchange and enhance clinical advice to SOPC and hospices. The gap is narrowed between primary palliative care and specialized neuropalliative care. The study investigates whether the intervention enables palliative patients to safely stay at home or in hospice for care, without an additional burden to them due to unnecessary hospitalizations because of neurological signs and symptoms.

### Limitations

The previous pilot study [33] demonstrated that neuropalliative teleconsultations are feasible. The TANNE trial builds on this approach and is therefore expected to succeed. Nevertheless, the calculated sample size may not be reached within the allotted time frame due to inclusion challenges, e.g. during the COVID 19 pandemic crisis. In addition, some data may not be completely collected because the study design is complex and requires attention from the participants, who are supported by the study personnel.

### Ethical considerations

The patients are all clients in palliative care and accordingly highly vulnerable. The teleconsultations and the accompanying data collections are time-consuming. Some questions may guide the patients’ attention to sensitive topics, potentially enhancing distress. For this reason, the patients are offered support in completing the questionnaires. All study participants may contact the consultants’ office if they desire any support. Subjects are advised to complete the questionnaires only to the extent that seems feasible to them. A broad time frame is indicated, during which participants can flexibly fill out the questionnaires, and participants are allowed to interrupt and continue answering as they wish.

Before participation, patients, relatives, legal representatives (when appointed), and palliative care teams receive information and consent forms, which describe aims, procedures, partners, data security measures, advantages, and disadvantages of the study. Participation is voluntary, and usual care is continued in case of a decision not to participate. Besides, AOK patients receive additional information, consent forms, and privacy statements regarding insurance data.

The study protocol was approved by the research ethics committee of the Ludwig-Maximilians-University LMU Munich (reference number: 20–1066).

### Data protection and data management

All participants are informed in advance about the storage and the use of the gathered data. Private data include all personal information available from the consultations and from the data management systems. Data are collected both electronically and in paper format, the latter being entered into the data management systems Pallidoc or ISPC and exported from there for further statistical analysis. The data reported on paper are collected at the study center in the hospital Krankenhaus Agatharied and stored inaccessibly for ten years after the end of the study before deletion. An electronic database is generated according to the requirements for the study. All changes made to the data are stored in an audit trail. The database is integrated into a general information technology infrastructure and security concept with firewall and backup system. The data are backed up on a regular basis.

The data from the data management systems are stored in a secure database in accordance with the internal data protection guidelines of the company and in compliance with German and European law. All data gathered during the study is integrated in this secure database. Data from patients and relatives are pseudonymized. A match between participants and pseudonyms is only possible with a table that is located at the consultantsʼ office. All data for evaluation is provided to select consortium partners only in a pseudonymized fashion.

### Impact

In summary, the TANNE trial aims at evaluating teleconsultations as part of a novel telemedicine-based intervention format for the highly specialized field of neuropalliative care on the quantitative, qualitative, and health economic levels, regarding objective outcome measures and subjective satisfaction in patients, relatives, and health care professionals, such as palliative care physicians as well as SOPC and hospice teams. A partially randomized, controlled trial is supposed to generate evidence for the meaningful and effective support by telemedicine-mediated expertise, such that health care services are improved and patients, with their caregivers, experience a better quality of life at the end of life.

## Data Availability

Data sharing is not applicable to this article as no datasets were generated or analyzed during the current study.

## Abbreviations

TANNE: “Telemedizinische Antworten auf Neuropalliative Nachfragen in Echtzeit” (in German), i.e. “Telemedical answers to neuropalliative inquiries in real-time”
SOPC: specialized outpatient palliative care (i.e. “SAPV: Spezialisierte ambulante Palliativversorgung” in Germany)
QOL: Quality of life
IPOS: Integrated Palliative Care Outcome Scale
McGill QOL-R: Revised McGill Quality of Life Questionnaire
ECOG: Eastern Cooperative Oncology Group Performance Status Scale
FAS-A: Full-analysis set A
FAS-C: Full-analysis set C
ES-A: Evaluation set A
ES-C: Evaluation set C
tES-A: Partial-evaluation set A
ISPC: Information system for palliative care
AOK: “Allgemeine Ortskrankenkasse”, i.e. a large statutory health insurance provider in Germany
